# Photoluminescence mechanisms of BF_2_-formazanate dye sensitizers: a theoretical study†

**DOI:** 10.1039/d4ra02240h

**Published:** 2024-06-24

**Authors:** Parichart Suwannakham, Pannipa Panajapo, Phorntep Promma, Tunyawat Khrootkaew, Anyanee Kamkaew, Kritsana Sagarik

**Affiliations:** a School of Chemistry, Institute of Science, Suranaree University of Technology Nakhon Ratchasima 30000 Thailand kritsana@sut.ac.th +66 81 8783994 +66 81 8783994

## Abstract

Photodynamic therapy (PDT) is an alternative, minimally invasive treatment for human diseases such as cancer. PDT uses a photosensitizer to transfer photon energy directly to cellular ^3^O_2_ to generate ^1^O_2_ (Type II), the toxicity of which leads to cancer cell death. In this work, the photoluminescence mechanisms of a BF_2_-formazanate dye sensitizer (BF_2_-FORM) and its iodinated derivative (BF_2_-FORM-D) were studied using complementary theoretical approaches; the photoluminescence pathways in the S_1_ and T_1_ states were studied using density functional theory (DFT) and time-dependent (TD)-DFT methods, the kinetic and thermodynamic properties of the pathways using the transition state theory (TST), and the time evolution and dynamics of key processes using non-adiabatic microcanonical molecular dynamics simulations with surface-hopping dynamics (NVE-MDSH). Evaluation of the potential energy surfaces (PESs) in terms of the rotations of the phenyl rings suggested a pathway for the S_1_ → S_0_ transition for the perpendicular structure, whereas two pathways were anticipated for the T_1_ → S_0_ transition, namely, [T_1_ → S_0_]_1_ occurring immediately after the S_1_/T_1_ intersystem crossing (ISC) and [T_1_ → S_0_]_2_ occurring after the S_1_/T_1_ ISC and T_1_ equilibrium structure relaxation, with the T_1_ → S_0_ energy gap being comparable to the energy required for ^3^O_2_ → ^1^O_2_. The PESs also showed that because of the heavy-atom effect, BF_2_-FORM-D possessed a significantly smaller S_1_/T_1_ energy gap than BF_2_-FORM. The TST results revealed that at room temperature, BF_2_-FORM-D was thermodynamically more favorable than the parent molecule. Analysis of the NVE-MDSH results suggested that the librational motions of the phenyl rings play an important role in the internal conversion (IC) and ISC, and the S_1_/T_1_ ISC and T_1_ → S_0_ transitions could be enhanced by varying the irradiation wavelength and controlling the temperature. These findings can be used as guidelines to improve and/or design photosensitizers for PDT.

## Abstract 2

Anti-L-ALM of the phenyl rings should be suppressed/delayed, whereas non-L-ALM should be promoted to enhance photoluminescence of the BF_2_-FORM-D photosensitizer.

## Graphical abstract

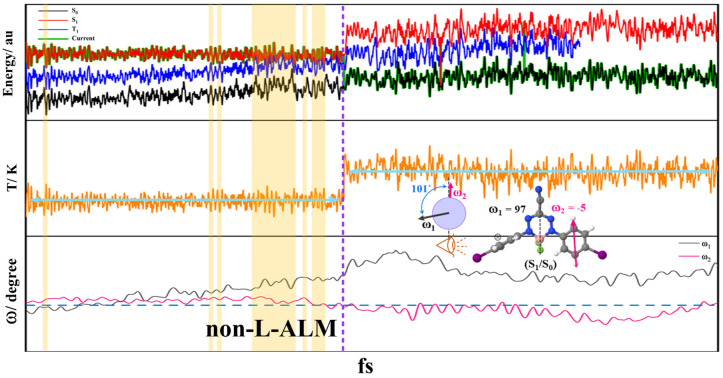

## Introduction

Photosensitization occurs when light at an appropriate wavelength interacts with a photosensitizer, from which the photon energy is transferred onto target molecules.^1^ Photodynamic therapy (PDT) is an alternative, minimally invasive treatment for various human diseases,^2,3^ including cancer, rheumatoid arthritis, and psoriasis, that uses only photon energy.^1^ PDT uses a photosensitizer that releases appropriate energy to generate singlet oxygen (^1^O_2_) from triplet oxygen (^3^O_2_).^4^ The cellular oxygen (^3^O_2_) absorbs the energy released from the photosensitizer to generate ^1^O_2_,^5^ and the toxicity of ^1^O_2_ leads to cell death in cancer, *e.g.*, for ^3^O_2_ (^3^∑_g_) → ^1^O_2_ (^1^Δ_g_), Δ*E*^T_1_→S_1_^ ∼ 0.97 eV (ref. 5) (∼94 kJ mol^−1^); in normal cells (healthy tissues), the oxygen levels are in general lower compared to cancer cells, thus resulting in less ^1^O_2_ generation and lower cytotoxic effects.^6^ Effective photosensitizers for therapeutics should have high light absorption coefficients, particularly in the infrared/near-infrared range, to allow for effective tissue penetration. They should exhibit low photobleaching quantum yields, high intersystem crossing (ISC) efficiencies, and low toxicity in the absence of light.^7^ These include the photodynamic properties, such as appropriate triplet relaxation energy (Δ*E*^T_1_→S_0_^), high phosphorescence quantum yield (*Φ*_T_), and long lifetime (*τ*^T_1_^) of the triplet state.^8^

**Fig. 1:**
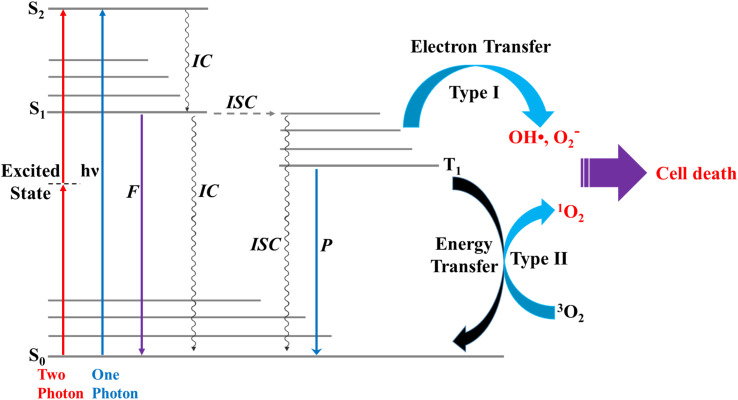
Schematic diagram showing two types of reaction pathways for formation of singlet oxygen from triplet oxygen, ^3^O_2_ (^3^∑_g_) → ^1^O_2_ (^1^Δ_g_). ISC = intersystem crossing; IC = internal conversion; F = fluorescence; P = phosphorescence.

The two photochemical mechanisms of PDT involving oxygen molecules are shown in Fig. 1. In Type I, radicals (*e.g.*, OH˙) act as intermediates, transferring electron energy from the photosensitizer to the oxygen derivatives, whereas for Type II, the photosensitizer passes light energy directly to the oxygen molecules.^4^ In general, phosphorescence is not easily observed because of the interference of fluorescence,^8^ and triplet excited states are difficult to generate through direct photoexcitation because ISC is symmetrically forbidden. For example, the S_1_ → T_1_ transition in chromophores possesses a large singlet–triplet energy gap.^9^

Theoretical and experimental studies have proposed several strategies to improve the efficiency of ISC and promote the generation of triplet states. These strategies include for example: Heavy atom effect;^10^ the introduction of heavy atoms like halogen (such as Br and I) into photosensitizers has been shown to increase ISC rates, and the introduction of chalcogen (such as S, Se, or Te) into the photosensitizers could also increase the production of ^1^O_2_ upon irradiation due to spin–orbit interactions:^11^ Molecular design;^12^ the modification of the structure of photosensitizer molecules can optimize their electronic characteristics to promote ISC and: Sensitizer–sensitizer interactions;^13^ ISC can also be enhanced through interactions among photosensitizer molecules, whether through complex formation or aggregation.

Boron difluoride (BF_2_) complexed with π-conjugated organic compounds is widely used as a photosensitizer in PDT, among which boron dipyrromethene (IUPAC = 4,4-difluoro-4-bora-3*a*,4*a*-diaza-*s*-indacene, or BODIPY) and its derivatives are the most widely studied.^14,15^ Such compounds have been reported to possess visible absorption and fluorescence emission between 470 and 550 nm, with a high emission quantum yield (*Φ*_F_ = 0.60).^16^ Although BODIPY has a high potential to be a functional component in PDT, attempts have been made to improve *Φ*_T_ using phenyl and/or heavy-atom substitutions.^8^

Several experimental methods have been developed to enhance the triplet excitation of photosensitizers by incorporating halogen atoms, such as the iodine (I) or bromine (Br) atoms, into organic aromatic compounds; these heavy atoms could help increase the spin–orbit coupling (SOC) and ISC rate, as well as the ^1^O_2_ quantum yield of photosensitizers.^17,18^ In most cases, the I atom has a higher ^1^O_2_ quantum yield (SOQY) than the Br atom.^19^ The higher *Φ*_T_ and SOQY for the I atom than the Br atom can be attributed to several factors, such as the higher heavy atom effect, higher radiative decay and lower internal conversion (IC) rates.^20–22^ These collective factors could lead to an enhancement of *Φ*_T_ and SOQY in BODIPY-based PDT photosensitizers.

**Fig. 2:**
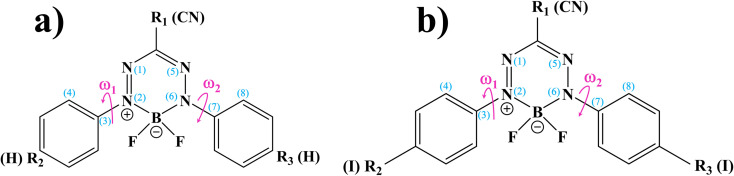
(a) and (b) Structures and numbering systems of BF_2_-FORM and BF_2_-FORM-D dye sensitizers, respectively. BF_2_-FORM = 3,3-difluoro-2,4-diphenyl-2,3-dihydro-1,2,4λ^4^,5,3λ^4^-tetrazaborinine; BF_2_-FORM-D = 3,3-difluoro-2,4-bis(4-iodophenyl)-2,3-dihydro-1,2,4λ^4^,5,3λ^4^-tetrazaborinine-6-carbonitrile; *ω*_1_ and *ω*_2_ = dihedral angles used in the calculations of the potential energy surfaces.

BF_2_-formazanate (3,3-difluoro-2,4-diphenyl-2,3-dihydro-1,2,4λ^4^,5,3λ^4^-tetrazaborinine, BF_2_-FORM in this work) is a fluorophore with several applications in microscopy. The structure of BF_2_-FORM, shown in Fig. 2, consists of a BF_2_ group coupled to a chelating N-donor ligand, forming a stable six-membered heterocyclic ring. BF_2_-FORM exhibits outstanding photophysical properties such as large molar extinction coefficients and high *Φ*_F_, which are generally in the far-red or near-infrared region. Therefore, BF_2_-FORM has significant potential in cell imaging and PDT applications.^23,24^

The optical properties of BF_2_-FORM are strongly affected by the electron-donating substituents at R_2_ and R_3_ (Fig. 2), leading to an increase in *Φ*_F_ and a red-shift in the emission spectra. The strong red-shift could be attributed to a smaller HOMO–LUMO energy gap upon the substitutions.^6^ For example, in the case of F-BODIPY, the introduction of two ethyl groups at the C_2_ and C_8_ positions led to a red-shifted absorption spectra compared to the parent molecule due to a decrease in the HOMO–LUMO energy gap and large charge transfer interaction within the molecule.^25^ Because electron-withdrawing substituents at R_1_ could also affect the photophysical properties, the differences in optical properties between Ph-, CN-, and NO_2_-substituted BF_2_-FORM are attributed primarily to the electron-withdrawing nature of the substituents (*e.g.*, NO_2_ > CN ≫ Ph).^26^

In our previous study,^24^ the photochemical properties of BF_2_-FORM-based photosensitizers were experimentally and theoretically studied in the electronic ground (S_0_), lowest singlet, and triplet excited states (S_1_ and T_1_) using density functional theory (DFT) and time-dependent density functional theory (TD-DFT) methods with the Becke, 3-Parameter, Lee–Yang–Parr (B3LYP) hybrid functional and 6-311G basis sets. A comparison of the experimental and theoretical results showed that the DFT/B3LYP/6-311G and TD-DFT/B3LYP/6-311G methods could provide insight into the PDT mechanisms and confirmed the effect of heavy-atom substituents (I and Br at R_2_ and R_3_) on the ISC rate.

In this study, complementary theoretical approaches were applied to investigate the photoluminescence mechanisms of BF_2_-FORM to improve its efficiency as a photosensitizer in PDT. Because theoretical and experimental studies^24^ revealed that substitutions of R_2_ and R_3_ at the phenyl rings by I and R_1_ at the heterocyclic ring by CN can significantly enhance the S_1_/T_1_ ISC with high *Φ*_T_, both BF_2_-FORM and its iodinated derivative [3,3-difluoro-2,4-bis(4-iodophenyl)-2,3-dihydro-1,2,4λ^4^,5,3λ^4^-tetrazaborinine-6-carbonitrile, BF_2_-FORM-D in this work] were selected as model molecules.

Theoretical studies focusing on Type II mechanism began with calculations of the equilibrium structures and energetic and spectroscopic properties of BF_2_-FORM and BF_2_-FORM-D in the S_0_, S_1_, and T_1_ states. The potential energy surfaces (PESs) for the S_1_ → S_0_, S_1_/T_1_, and T_1_ → S_0_ transitions were computed using the nudged elastic band (NEB) method, from which the kinetics and thermodynamics of the photoluminescence pathways were studied using the transition state theory (TST). Emphasis was placed on the probabilities of IC and ISC, as well as on the effect of the electron-withdrawing substituents on the photophysical properties. Furthermore, to explore the possibility of increasing photoluminescence, non-radiative relaxation, and effect of molecular dynamics on the S_1_ → S_0_ transition and S_1_/T_1_ ISC were studied using non-adiabatic microcanonical molecular dynamics simulations with surface-hopping dynamics (NVE-MDSH). The theoretical results are discussed in comparison with available theoretical and experimental data.

## Computational methods

### Quantum chemical calculations

All the DFT and TD-DFT calculations were performed using the TURBOMOLE 7.50 software package.^27^ For the TD-DFT method, the Tamm–Dancoff approximation (TDA) was applied to avoid singlet instabilities in the lowest singlet and triplet state calculations. The DFT and TD-DFT methods with the B3LYP functionals were chosen based on several benchmarking calculations in photochemical reactions,^28,29^ including BODIPY-based photosensitizers.^9^ For example, our benchmarks against the complete active space multiconfigurational second-order perturbation theory (CASPT2) method revealed that for the photodissociation and formation of glycine,^28,29^ the characteristic structures and energies on the S_0_ and S_1_ PESs obtained from the DFT/B3LYP and TD-DFT/B3LYP methods were in good agreement with the CASPT2 results, and DFT/B3LYP/6-311G calculations on the I2-IR783-Mpip photosensitizer^6^ revealed the equilibrium structures, energetics and absorption spectra comparable with experimental data; the 6-311G basis set was also used successfully in a theoretical study on photoinduced charge separation-charge recombination in BODIPY compounds.^30^ The performance of the DFT/B3LYP method with various sizes of the basis sets was discussed in detail using boron-doped triazine based covalent organic framework as a model molecule in ref. 31.

#### Equilibrium structures

**Fig. 3:**
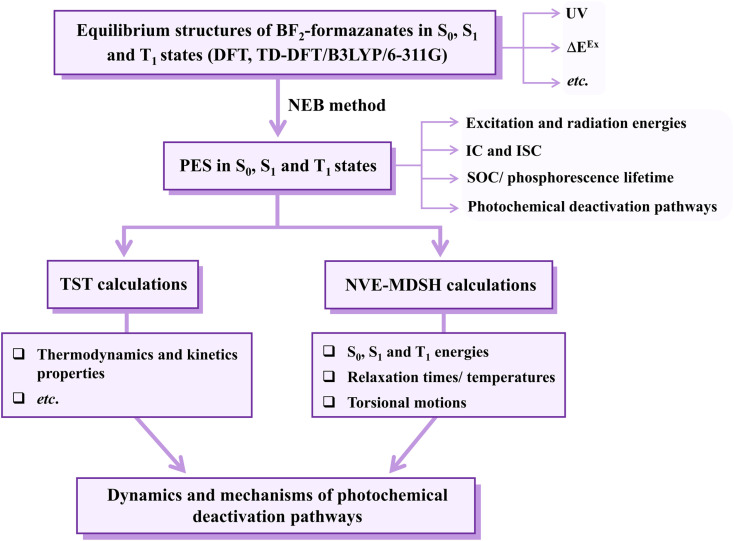
The theoretical strategy and methods used to study photoluminescence mechanisms of the BF_2_-formazanate dye sensitizers. PES = potential energy surface; Δ*E*^Ex^ = excitation energy; IC and ISC = internal conversion and intersystem crossing; TST = transition state theory; NVE-MDSH = non-adiabatic microcanonical molecular dynamics simulations with surface hopping dynamics.

To study photoluminescence pathways for BF_2_-FORM and BF_2_-FORM-D, the theoretical strategy and methods shown in Fig. 3 were used. Calculations of the equilibrium structures, energetics, and spectroscopic properties of the S_0_, S_1_, and T_1_ states were performed using the DFT/B3LYP/6-311G and TD-DFT/B3LYP/6-311G methods. The calculated equilibrium structures and energies of BF_2_-FORM and BF_2_-FORM-D in the S_0_, S_1_, and T_1_ states are listed in Tables S1 and S2,† respectively. The absorption spectra and fluorescence lifetimes of BF_2_-FORM and BF_2_-FORM-D were computed using the NEWTON-X software package^32–34^ interfaced with TURBOMOLE 7.50, for which 200 Wigner-sampled structures were used as initial conditions. The results were used as guidelines in the photoluminescence pathway analysis.

#### Reaction pathway optimizations

**Fig. 4:**
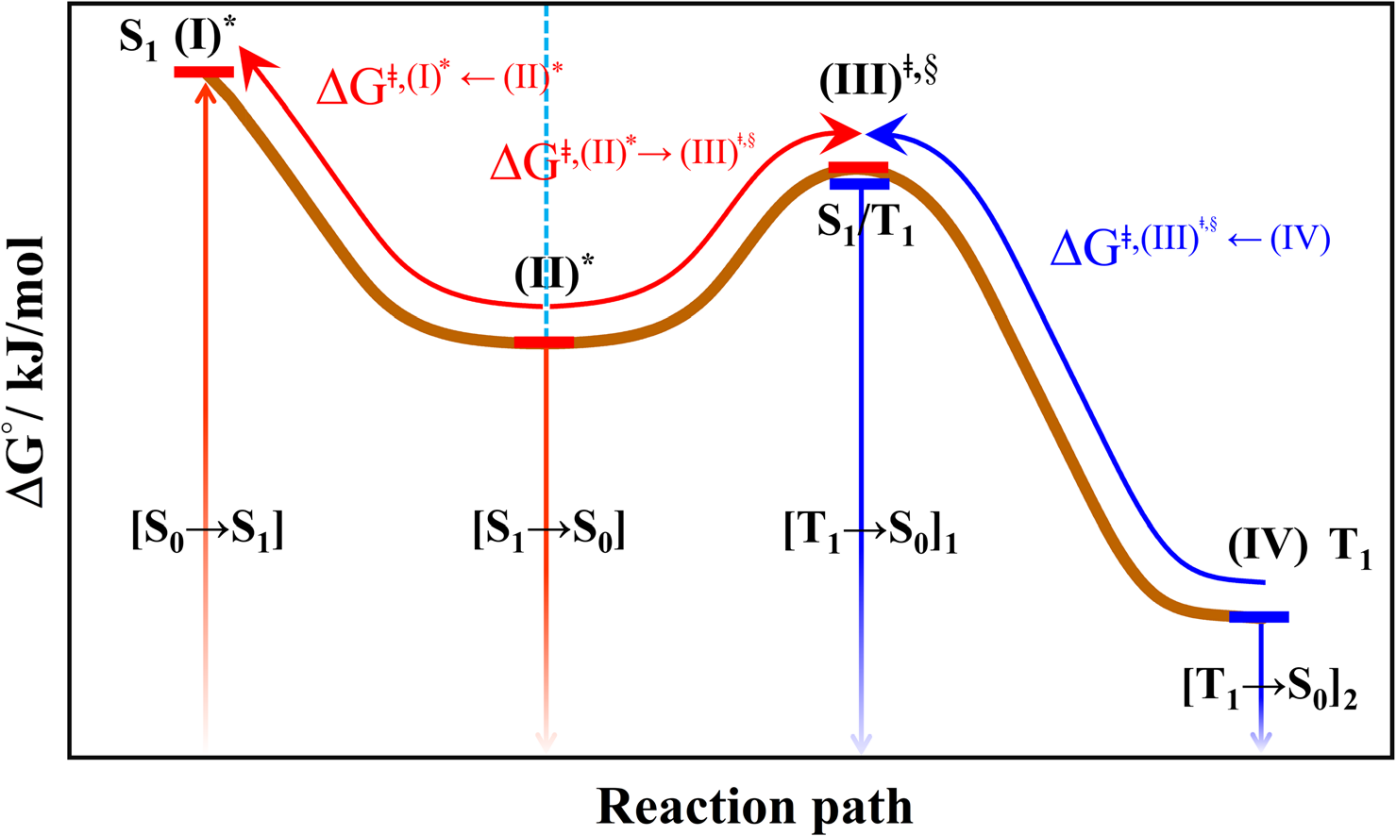
The hypothesized photochemical deactivation pathways ((I)* → (II)* ⇌ (III)^‡,§^ → (IV)) used in the present study.

The photoluminescence pathways were hypothesized in this work to start with an S_0_ → S_1_ vertically excited structure (I)*, as shown in Fig. 4, followed by (I)* → (II)* structural relaxation and S_1_ → S_0_ transition ([S_1_ → S_0_]). Two pathways were hypothesized for T_1_ → S_0_ transition, namely, (I)* → (II)* ⇌ (III)^‡,§^ and S_1_/T_1_ ISC followed by T_1_ → S_0_ transition ([(T_1_ → S_0_)]_1_) or (I)* → (II)* ⇌ (III)^‡,§^ and S_1_/T_1_ ISC followed by (III)^§^ → (IV) structural relaxation in the T_1_ state and T_1_ → S_0_ transition ([(T_1_ → S_0_)]_2_). These results led to the following three deactivation pathways:

1 (I)* → (II)* → [S_1_ → S_0_]

2 (I)* → (II)* ⇌ (III)^‡,§^ → S_1_/T_1_ ISC → [T_1_ → S_0_]_1_

3 (I)* → (II)* ⇌ (III)^‡,§^ → S_1_/T_1_ ISC → (IV) → [T_1_ → S_0_]_2_

The equilibrium structures of BF_2_-FORM and BF_2_-FORM-D in the S_0_, S_1_, and T_1_ states obtained from DFT/B3LYP/6-311G and TD-DFT/B3LYP/6-311G geometry optimizations were used in the PES calculations.

Theoretical studies have shown that ISC in aromatic organic compounds can be mediated by intramolecular motions^35^ such as molecular rotation or twist;^36^ thus, reaction pathway optimization began with the S_0_ → S_1_ vertically excited precursor (I)*, from which the PESs for the rotations of the torsional angles *ω*_1_ and *ω*_2_ (Fig. 2) were constructed. Reaction pathway optimization was performed using the NEB method^37^ with limited-memory Broyden–Fletcher-Goldfarb-Shanno (LBFGS) optimizers included in the ChemShell software package.^38^ In this work, to search for minimum energy pathways connecting the initial and final structures on the PESs, approximately 10 intermediate images (including the saddle points) along the pathways were optimized. In the NEB calculations,^37^ the gradients on the reaction pathway were calculated based on the spring forces acting on local tangents between each image and on the true forces acting perpendicular to the local tangents.

Because SOC plays an important role in ISC and phosphorescence, the singlet–triplet energy gaps along the S_1_ PES were computed using the TD-DFT/B3LYP/6-311G method and the geometries obtained from the NEB calculations. The energy gaps were refined using the RICC2/aug-cc-pVDZ method, from which the phosphorescence lifetimes were computed. SOC was computed using TURBOMOLE 7.50 based on the effective spin-orbital mean field approximation,^39^ in which the mean field two-electron contribution was computed from the Hartree–Fock density.

### Kinetics and thermodynamics of reaction pathways

To study the kinetics and thermodynamics of the proposed photoluminescence pathways, quantized vibrational rate constants (*k*^Q-vib^) were calculated over a temperature range of 300–550 K. This range of temperatures includes the standard human body temperature of 310 K. The *k*^Q-vib^ values were computed using eqn (4),^40^ in which Δ*E*^‡,ZPC^ is the zero-point energy-corrected barrier, obtained by including the zero-point correction energy (Δ*E*^ZPE^) to the energy barriers obtained from the NEB method (Δ*E*^‡^).4
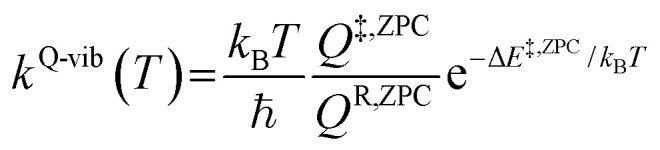
where *Q*^R,ZPC^ and *Q*^‡,ZPC^ are the partition functions of the precursor and transition structures, respectively, and *k*_B_ and *ℏ* are the Boltzmann and Planck constants, respectively. The activation free energies (Δ*G*^‡^) were derived from *k*^Q-vib^ using *k*^Q-vib^ (*T*) = (*k*_B_*T*/ℏ)e^−Δ*G*^‡^^/^*RT*^, and the activation enthalpies (Δ*H*^‡^) were computed using eqn (5).5
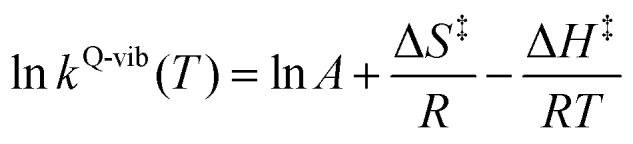
where Δ*S*^‡^ is the activation entropy and *R* is the gas constant. The value of Δ*H*^‡^ was obtained from the linear relationship between ln *k*^Q-vib^ and 1000/*T*.

Based on the photoluminescence pathways shown in Fig. 4, the thermodynamics of the consecutive reaction pathway (I)* → (II)* ⇌ (III)^‡,§^ → (IV) were studied, in which (II)* ⇌ (III)^‡,§^ → (IV) was assumed to be in a quasi-equilibrium. The thermodynamic property of interest was the total Gibbs free energy (Δ*G*°^,tot^) of the reactions, computed using Δ*G*^‡^ obtained from the TST method. Δ*G*°^,tot^ was computed by dividing the consecutive reaction pathway into two single steps, namely, (I)* → (II)* and (II)* ⇌ (III)^‡,§^ → (IV). For (I)* → (II)*, 
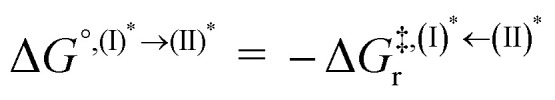
, whereas 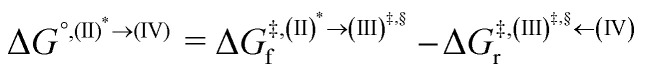 for (II)* ⇌ (III)^‡,§^ → (IV) and Δ*G*^°,tot^ = Δ*G*^°,(I)*→(II)*^ + Δ*G*^°,(II)*→(IV)^. All the kinetic and thermodynamic properties were computed using the DL-FIND program^41^ included in the ChemShell software package.^38^

Because the (I)* → (II)* relaxation on the S_1_ PES is exothermic (Δ*H*° < 0) and thermal energy is required for (II)* ⇌ (III)^‡,§^ → (IV), the thermodynamic spontaneity could be studied. The spontaneous temperature (*T*_s_), below which the transition structure (III)^‡,§^ at the S_1_/T_1_ intersection is spontaneously formed from (I)*, was obtained from the plot of 
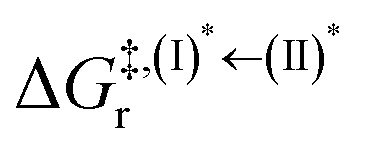
 and 
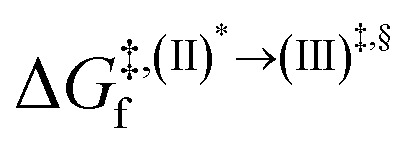
*versus* temperature; (I)* → (II)* ⇌ (III)^‡,§^ is spontaneous when 
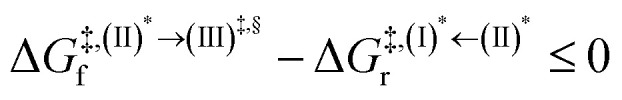
. In other words, the formation of (III)^‡,§^ is spontaneous when *T* ≤ *T*_s_.

### Surface-hopping molecular dynamics simulations

Because non-radiative relaxation reduces the emission quantum efficiency, to study the non-radiative S_1_ → S_0_ relaxation in BF_2_-FORM-D, NVE-MDSH simulations were performed. Because the calculations were computationally intensive, NVE-MDSH simulations were conducted using DFT and TD-DFT methods with a smaller (DZP) basis set, except for the iodine atoms, for which the larger TZVPall basis set^42^ was used to consider the heavy-atom effect. Fifty initial configurations were generated based on the Wigner distribution, from which NVE-MDSH simulations were performed over a time span of ∼4 ps using the TURBOMOLE 7.50 software package.

The integration of Newton's equations of motion was conducted using the Verlet algorithm with a timestep of 0.5 fs, which was confirmed in our previous studies to be sufficient to study photochemical processes.^28^ The characteristic dynamics in the S_1_ states were categorized, and representative reactions were chosen and investigated in detail. To study the possibility of increasing the S_1_/T_1_ ISC, irradiation wavelengths, intramolecular motions, and temperatures, as well as the probabilities for the S_1_/T_1_ ISC and T_1_ → S_0_ transition, were analyzed in detail for BF_2_-FORM-D.

## Results and discussion

To discuss characteristic structures of BF_2_-FORM and BF_2_-FORM-D, a three-character code is used, *e.g.*, G-[*k*]^eq^, ^1^E-[*k*]^‡^, or ^3^E-[k]^§^, where G indicates the structure in the S_0_ state and ^1^E and ^3^E indicate the structures in the S_1_ and T_1_ states, respectively. The terms […]^eq^ and […]* denote the equilibrium and vertically excited structures, respectively, whereas […]^‡^ and […]^§^ represent the transition structure and structure at the S_1_/T_1_ intersection, respectively. Different structures on the same PES are labeled as [*k*]. For example, ^1^E-[1]* and ^1^E-[2]^eq^ represent two structures on the S_1_ PES. Additional symbols are used to represent the characteristic energies on the PES. In the discussion, for example, Δ*E*^S_0_→S_1_^ and Δ*E*^‡^ represent the S_0_ → S_1_ vertical excitation energy and energy barrier on PES, respectively, whereas Δ*E*^S_1_/T_1_^ represents the energy gap between the S_1_ and T_1_ states at or in the vicinity of the S_1_/T_1_ intersection. The terms (…)^S_0_→S_1_^, (…)^‡^ and (…)^S_1_/T_1_^ are used to represent the corresponding energies in the figures.

### Static properties

#### Equilibrium structures

**Fig. 5:**
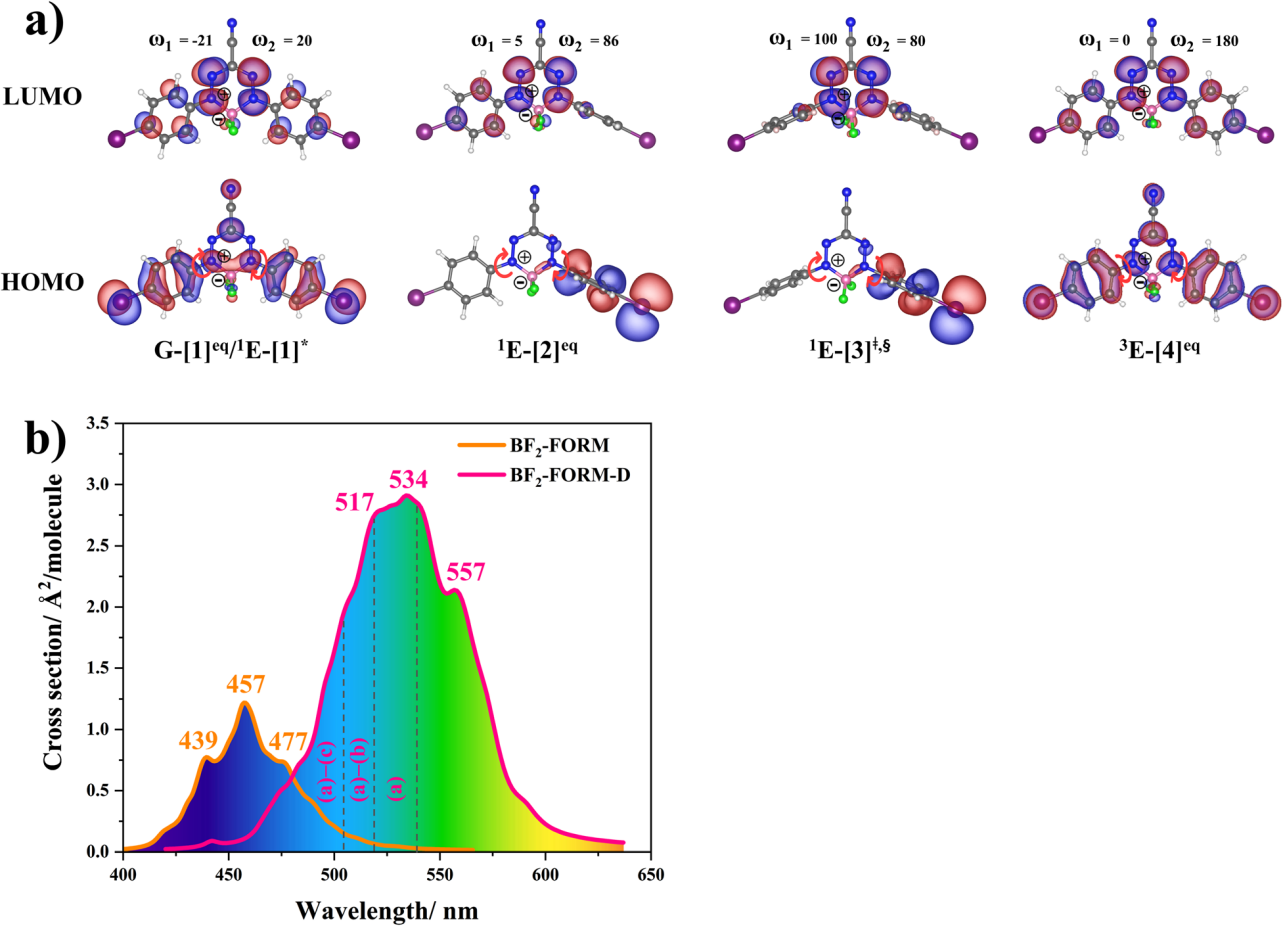
(a) Structures of BF_2_-FORM-D in the S_0_, S_1_ and T_1_ states, obtained based on DFT/B3LYP/6-311G and TD-DFT/B3LYP/6-311G calculations. The code symbols are explained in the text. (b) Absorption spectra obtained based on 200 Wigner sampled structures.

The equilibrium structures and energies of BF_2_-FORM and BF_2_-FORM-D in the S_0_, S_1_, and T_1_ states obtained from DFT/B3LYP/6-311G and TD-DFT/B3LYP/6-311G geometry optimizations are presented in Tables S1 and S2,† respectively. Because the equilibrium structures and highest occupied molecular orbital-lowest unoccupied molecular orbital (HOMO–LUMO) of BF_2_-FORM and BF_2_-FORM-D were not significantly different, only the results for BF_2_-FORM-D are shown in Fig. 5.

The equilibrium structures of BF_2_-FORM and BF_2_-FORM-D in the S_0_ and T_1_ states were virtually identical, as shown by the bent and perfect planar structures G-[1]^eq^ and ^3^E-[4]^eq^, respectively, in Fig. 5a. The HOMOs of G-[1]^eq^ and ^3^E-[4]^eq^ were characterized by a strong π character in the formazanate heterocyclic and phenyl rings, whereas the electron density of the LUMO was highly localized, resulting in a significantly lower degree of conjugation in the S_1_ and T_1_ states.

The equilibrium structures of BF_2_-FORM and BF_2_-FORM-D in the S_1_ state were quite different. BF_2_-FORM was represented by a propeller structure (Fig. S1†) or a twisted bent structure with the same HOMO–LUMO as G-[1]^eq^, whereas BF_2_-FORM-D was represented by a perpendicular structure, ^1^E-[2]^eq^ (Fig. 5a). For ^1^E-[2]^eq^, the electron density distributions on the phenyl rings were not symmetrical because of the positive charge on the N(2) atom of the formazanate heterocyclic ring. Comparison of the vertical excitation energies of BF_2_-FORM and BF_2_-FORM-D in Tables S_1_ and S_2_† shows that the iodine substitutions at the phenyl rings directly affected Δ*E*^S_0_→S_1_^, namely, a strong red-shift is observed for BF_2_-FORM-D because of a more extensive electron density distribution in HOMO (G-[1]^eq^) and large charge transfer interaction within the molecule.

For BF_2_-FORM-D, Δ*E*^S_0_→S_1_^ = 2.42 eV (*λ*^S_0_→S_1_^ = 513 nm) was in good agreement with the absorption spectra obtained based on 200 Wigner-sampled structures, 
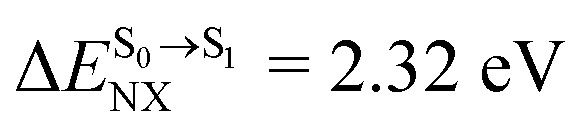
 (
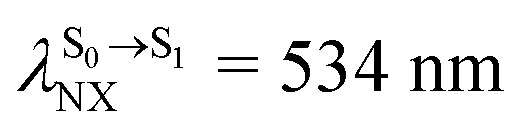
, shown in Fig. 5b) with the fluorescence lifetime, 
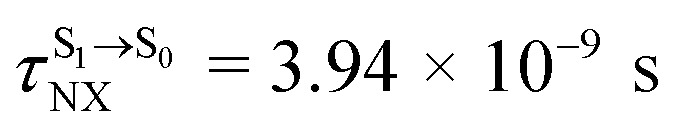
 (Table S2†). The RICC2/aug-cc-pVDZ results confirmed the bent structure (G-[1]^eq^) to possess 
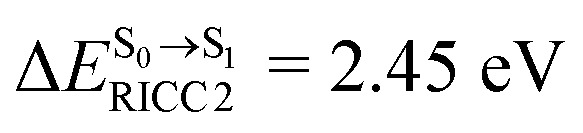

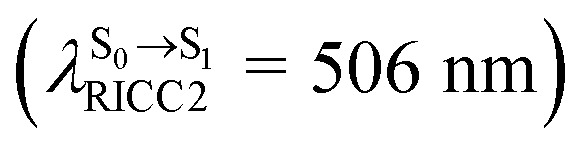
. The calculated excitation energies/wavelengths were in good agreement with the experimental absorption spectra (*e.g.*, in CHCl_3_, 
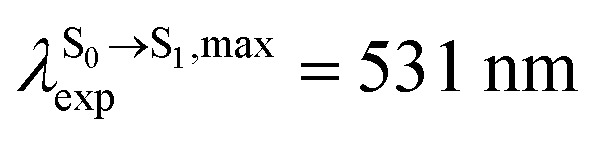
).^24^ For BF_2_-FORM, the vertical excitation energies were higher, where Δ*E*^S_0_→S_1_^ = 2.88 eV (*λ*^S_0_→S_1_^ = 430 nm) and 
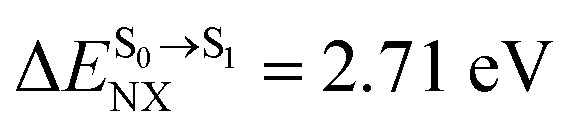
 (
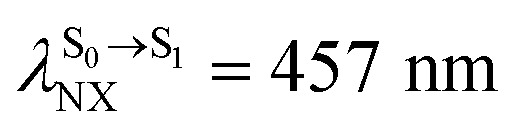
, shown in Fig. 5b) with 
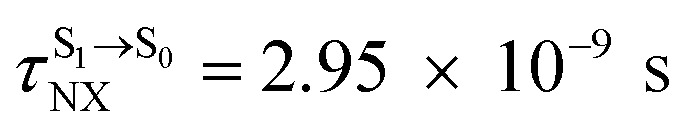
 (Table S1†). The energy values were compatible with the RICC2/aug-cc-pVDZ results, 
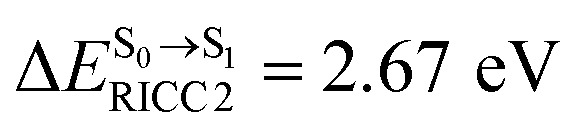
 (
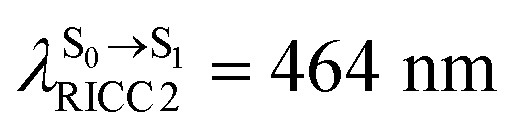
). The trend of 
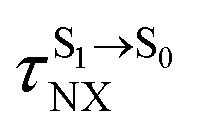
 was in good agreement with the experiment,^24^*e.g.*, in toluene, 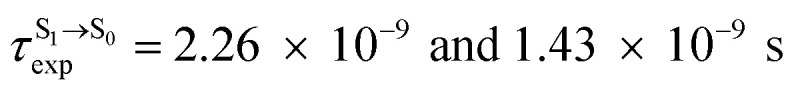 for BF_2_-FORM-D and BF_2_-FORM, respectively.

#### Potential energy surfaces

**Fig. 6:**
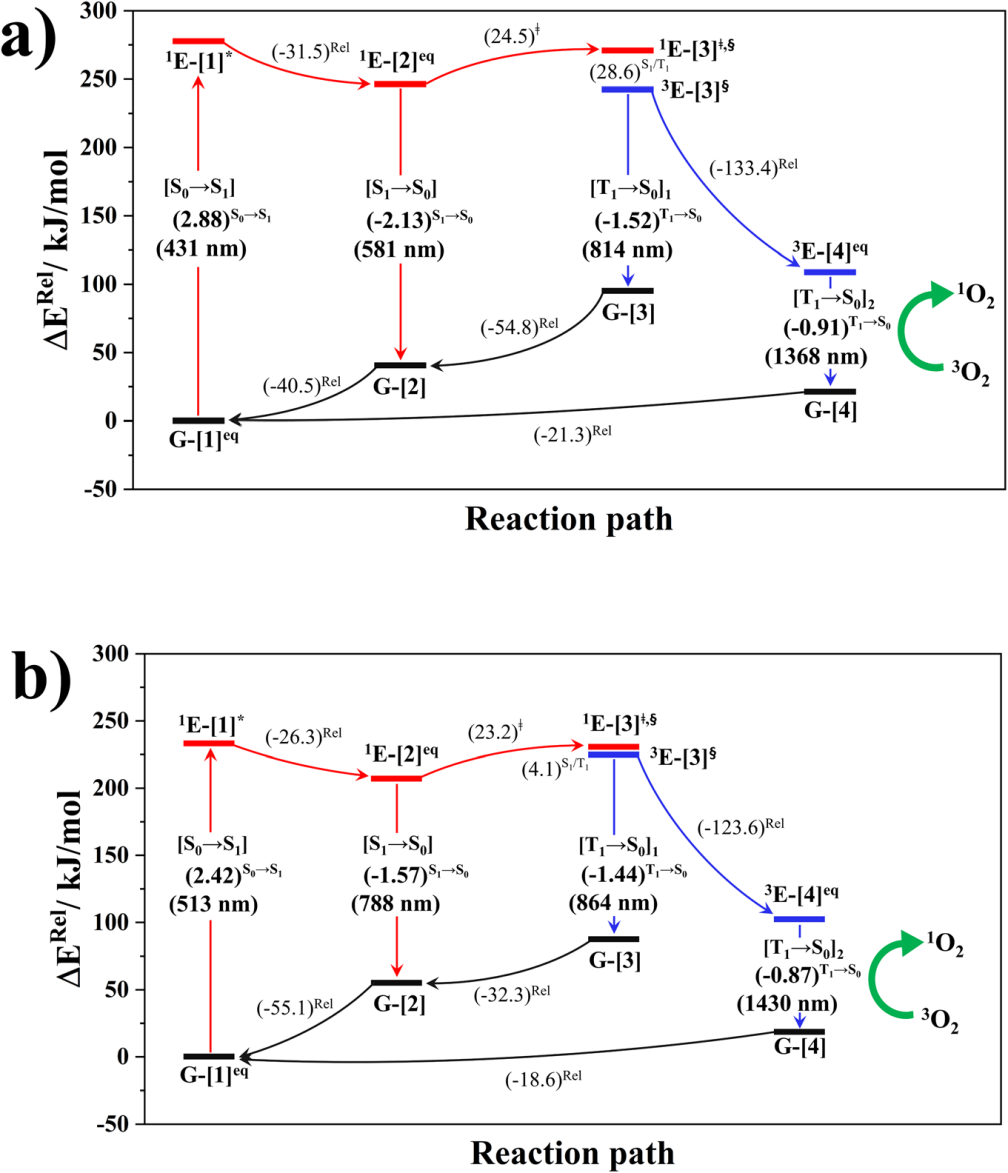
(a) and (b) Proposed photoluminescence pathways for BF_2_-FORM and BF_2_-FORM-D dye sensitizers obtained from the DFT/B3LYP/6-311G, TD-DFT/B3LYP/6-311G and NEB methods. Energies are in kJ mol^−1^ unless specified otherwise. (…)^S_0_→S_1_^ = vertical excitation energy in eV; Δ*E*^Rel^ = relative energy with respect to the total energy in the S_0_ state; (…)^Rel^ = relative energy with respect to the transition structure; (…)^‡^ = energy barrier; […]* = S_0_ → S_1_ vertically excited structure; […]^§^ = structure at the S_1_/T_1_ intersection.

The PESs for the rotations of the dihedral angles *ω*_1_ and *ω*_2_ in BF_2_-FORM and BF_2_-FORM-D, which were obtained using the DFT/B3LYP/6-311G, TD-DFT/B3LYP/6-311G, and NEB methods, are shown in Fig. S1 and S2,† respectively. Analysis of the PESs, shown in Fig. 6, revealed three potential deactivation pathways in which a possibility of the S_1_ → S_0_ transition ([S_1_ → S_0_]) is seen for the propeller structure of BF_2_-FORM with Δ*E*^S_1_→S_0_^ = −2.13 eV (*λ*^S_1_→S_0_^ = 581 nm), whereas Δ*E*^S_1_→S_0_^ = −1.57 eV (*λ*^S_1_→S_0_^ = 788 nm) is for the perpendicular structure (^1^E-[2]^eq^) of BF_2_-FORM-D. The former is the same as the experimental emission spectra of BF_2_-FORM, 
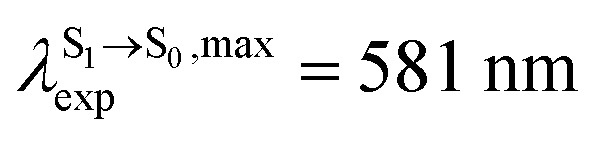
 in CHCl_3_, whereas the latter is within the range observed experimentally, 
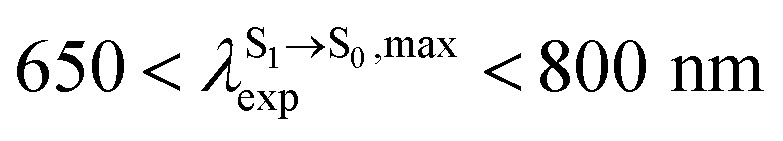
 for BF_2_-FORM-D.^24^

The photoluminescence pathways for BF_2_-FORM and BF_2_-FORM-D shown in Fig. 6 further suggest a possibility of the S_1_/T_1_ ISC at ^1^E-[3]^‡,§^, with the energy barriers for ^1^E-[2]^eq^ → ^1^E-[3]^‡,§^, Δ*E*^‡^ = 24.5 and 23.2 kJ mol^−1^, and S_1_/T_1_ energy gaps, Δ*E*^S_1_/T_1_^ = 0.30 and 0.04 eV, respectively. Because Δ*E*^S_0_→S_1_^ of BF_2_-FORM-D is closer to the center of the visible light spectrum (550 nm) with a smaller Δ*E*^S_1_/T_1_^, BF_2_-FORM-D is anticipated to be a more effective luminophore, mainly because of the heavy-atom effect. Therefore, subsequent discussions focus on BF_2_-FORM-D, with the results for BF_2_-FORM included in parentheses.

For BF_2_-FORM-D, two deactivation pathways for T_1_ → S_0_ transition, [T_1_ → S_0_]_1_ and [T_1_ → S_0_]_2_ in eqn (2) and (3), were observed after the S_1_/T_1_ ISC (Fig. 6). [T_1_ → S_0_]_1_ occurred immediately after the S_1_/T_1_ ISC, ^3^E-[3]^§^ → G-[3] with Δ*E*^T_1_→S_0_^ = −1.44 (−1.52) eV (*λ*^T_1_→S_0_^ = 864 (814) nm), whereas [T_1_ → S_0_]_2_ occurred after the ^3^E-[3]^§^ → ^3^E-[4]^eq^ structural relaxation in the T_1_ state, ^3^E-[4]^eq^ → G-[4] with Δ*E*^T_1_→S_0_^ = −0.87 (−0.91) eV (*λ*^T_1_→S_0_^ = 1430 (1368) nm). To confirm Δ*E*^T_1_→S_0_^ obtained from the TD-DFT/B3LYP/6-311G method and to study the phosphorescence lifetimes, RICC2/aug-cc-pVDZ calculations based on the spin–orbit coupling with perturbation theory (SOC-PT-CC2) were made on ^3^E-[3]^§^ for [T_1_ → S_0_]_1_ and on ^3^E-[4]^eq^ for [T_1_ → S_0_]_2_. The values obtained for [T_1_ → S_0_]_1_ are 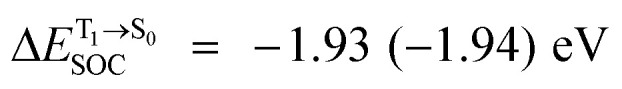 [*λ* = 642 (639) nm] and 
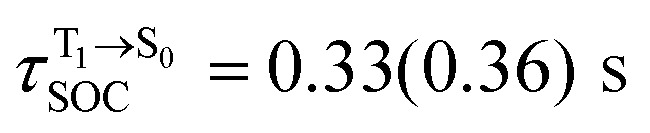
, whereas 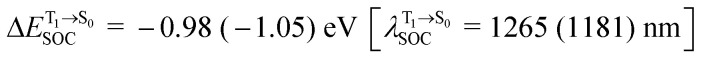 and 
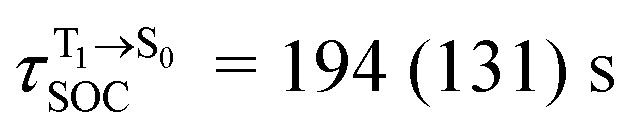
 for [T_1_ → S_0_]_2_.

Because Δ*E*^T_1_→S_0_^ and 
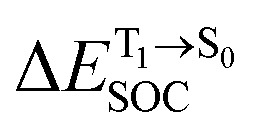
 for [T_1_ → S_0_]_2_ are close to the absorption energy for ^3^O_2_ (^3^∑_g_) → ^1^O_2_ (^1^Δ_g_), Δ*E*^T_1_→S_1_^ ≈ 0.97 eV,^5^ and 
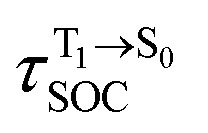
 is considerably longer than [T_1_ → S_0_]_1_, [T_1_ → S_0_]_2_ is confirmed to be a key process to drive ^3^O_2_ (^3^∑_g_) → ^1^O_2_ (^1^Δ_g_), and BF_2_-FORM-D is thus a better photosensitizer in PDT; experiments have shown that long-lived triplet excited state could promote the formation of ^1^O_2_ and increases the efficiency of the PDT.^11^ Therefore, only the kinetics and thermodynamics of [T_1_ → S_0_]_2_ are discussed further.

#### Thermodynamics and kinetics of the T_1_ → S_0_ transition

The kinetic and thermodynamic results for [T_1_ → S_0_]_2_ are shown in Tables S3 and S4† for BF_2_-FORM and BF_2_-FORM-D, respectively. Because ^1^E-[1]* → ^1^E-[2]^eq^ are barrierless, ^1^E-[2]^eq^ → ^1^E-[3]^‡,§^ could be considered the rate-determining step of the S_1_/T_1_ ISC. The TST results showed that BF_2_-FORM was kinetically more favorable than BF_2_-FORM-D; for example, at 300 K, 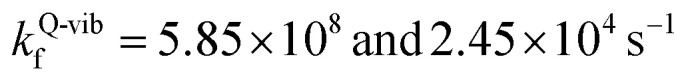, respectively.

**Fig. 7:**
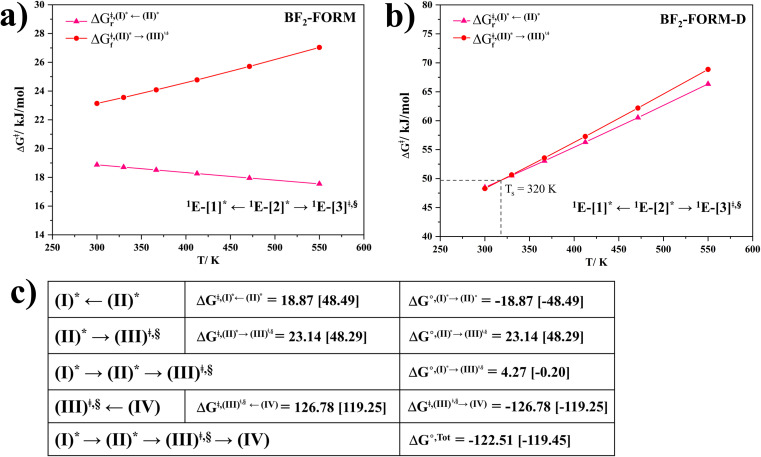
(a) and (b) Plots of 
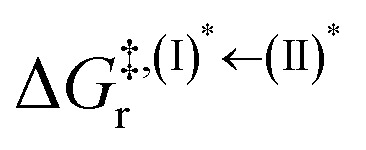
 and 
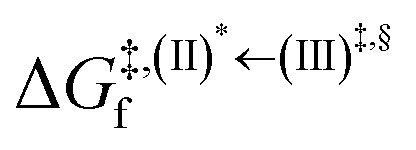
 as a function of *T* for BF_2_-FORM and BF_2_-FORM-D, respectively. *T*_s_ = spontaneous temperature below which the reaction is spontaneous. (c) Gibbs free energies for the photochemical deactivation processes (Fig. 4) for BF_2_-FORM at 300 K. […] = values for BF_2_-FORM-D.

Analysis of Δ*G*^°,tot^ in Fig. 7c shows that although [T_1_ → S_0_]_2_ was thermodynamically favorable for both BF_2_-FORM-D and BF_2_-FORM, *e.g.*, at 300 K, Δ*G*°^,tot^ = −119.5 and −122.5 kJ mol^−1^, respectively, the rate-determining process (II)* ⇌ (III)^‡,§^ (^1^E-[2]^eq^ → ^1^E-[3]^‡,§^) was spontaneous at room temperature only for BF_2_-FORM-D; for BF_2_-FORM-D, the plot of 
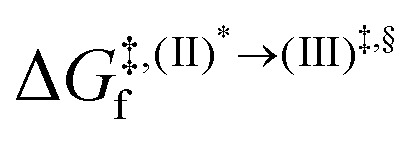
 and 
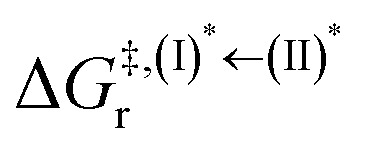
 as a function of *T* (Fig. 7b) showed that (I)* → (II)* ⇌ (III)^‡,§^ could be spontaneous at *T* ≤ *T*_s_ = 320 K, whereas the same plot did not show *T*_s_ for BF_2_-FORM (Fig. 7a). These results indicate that for [T_1_ → S_0_]_2_, although BF_2_-FORM was kinetically more favorable, BF_2_-FORM-D was thermodynamically more favorable because the ^1^E-[2]^eq^ → ^1^E-[3]^‡,§^ could be spontaneous below *T* = 320 K.

### Surface-hopping dynamics

**Fig. 8:**
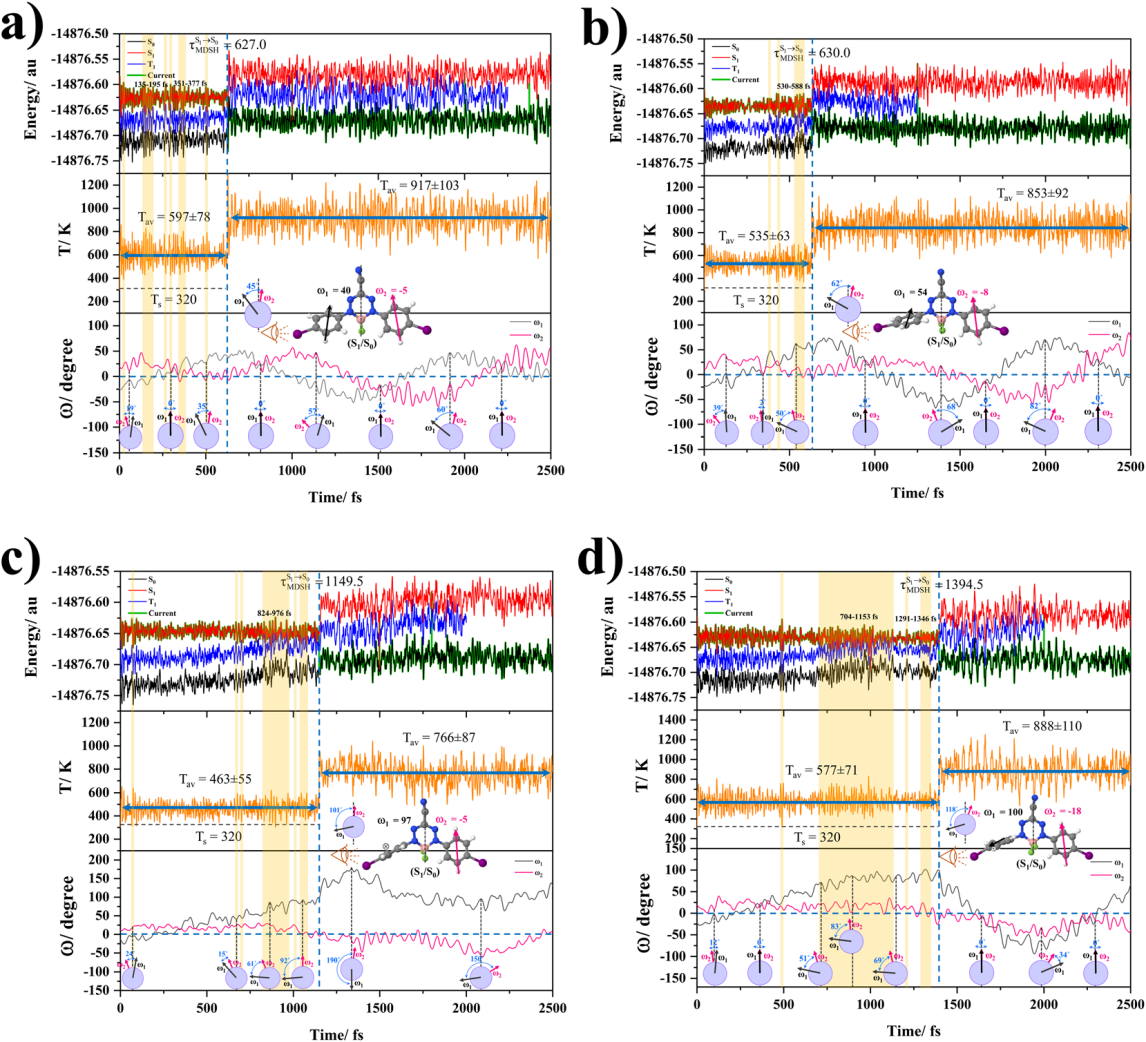
Examples of time evolutions of energies in the S_0_, S_1_ and T_1_ states, temperatures, and dihedral angles *ω*_1_ and *ω*_2_, obtained from NVE-MDSH simulations on BF_2_-FORM-D. 
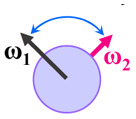
 The Newman projections at the bottom of the figures are used to differentiate the anti- and non-synchronous large-amplitude librational motions, anti-L-ALM and non-L-ALM, respectively. (a) and (b) anti-L-ALM. (c) and (d) non-L-ALM. 
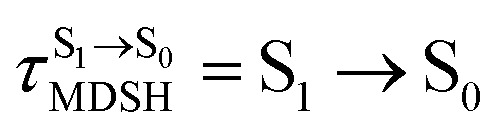
 surface hopping time; *T*_av_ = the average temperature; *ω*_1_ and *ω*_2_ = dihedral angles; *T*_s_ = spontaneous temperature; (S_1_/S_0_) = structure at 
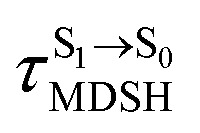
.

In order to enhance the photoluminescence quantum efficiency, it is important to study the non-radiative S_1_ → S_0_ relaxation process in BF_2_-FORM-D and the possibility of increasing the S_1_/T_1_ ISC. Because the NVE-MDSH simulations applied in this work considered only the non-radiative S_1_ → S_0_ relaxation ([S_1_ → S_0_] in Fig. 6), to explore the possibility of the S_1_/T_1_ ISC (^1^E-[3]^‡,§^ → ^3^E-[3]^§^ in Fig. 6), the time evolutions of the total energies in the S_0_, S_1_, and T_1_ states, temperatures, and dihedral angles *ω*_1_ and *ω*_2_ were extracted from the NVE-MDSH simulations and considered in the dynamic analysis. Characteristic results were selected as examples and are shown in Fig. 8.

The time evolutions of *ω*_1_ and *ω*_2_ suggest two characteristic motions of the phenyl rings; large- (L-ALM) and small-amplitude librational motions (S-ALM). L-ALM is characterized by *ω*_1_ and *ω*_2_ varying over a wide range, whereas S-ALM can be considered fine structures of L-ALM. To study the effect of L-ALM on the non-radiative S_1_ → S_0_ relaxation and on the S_1_/T_1_ ISC, two vectors were defined on the phenyl rings. Newman projections of these two vectors and Δ*ω*_MDSH_ = |*ω*_1,MDSH_ − *ω*_2,MDSH_| acquired from the NVE-MDSH simulations are shown in Fig. 8.

For BF_2_-FORM-D, the Newman projections and Δ*ω*_MDSH_ reveal two types of L-ALM, namely, anti- and non-synchronous L-ALM, abbreviated anti-L-ALM and non-L-ALM, respectively. Because the librational motions of *ω*_1_ and *ω*_2_ are coupled in the S_1_ state, anti-L-ALM is characterized by 
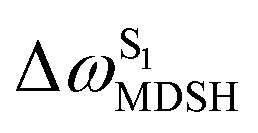
 varying uniformly across a narrow range, *e.g.*, 
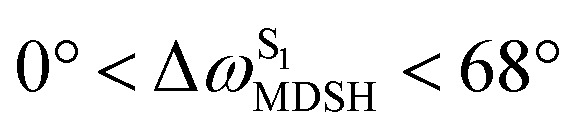
, shown in Fig. 8a and b, whereas 
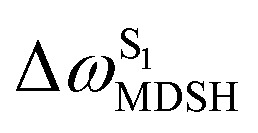
 for non-L-ALM varies across a wider range, *e.g.*, 
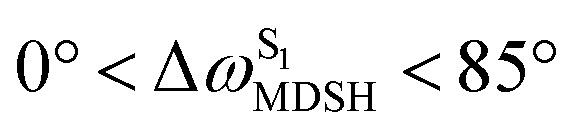
, shown in Fig. 8c and d. Analysis of the NVE-MDSH results shows that short S_1_ → S_0_ surface-hopping times 
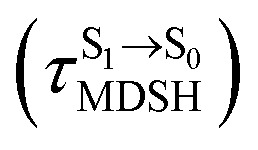
 are associated with anti-L-ALM (*e.g.*, 
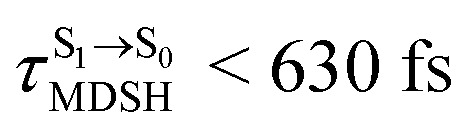
, shown in Fig. 8a and b), whereas non-L-ALM dominates for long 
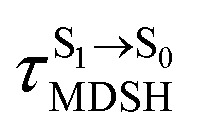
 (*e.g.*, 
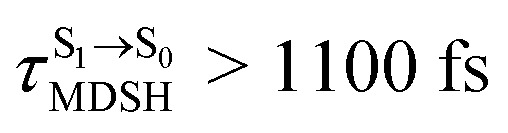
, shown in Fig. 8c and d).

Because the NVE-MDSH simulations do not directly account for S_1_ → S_0_ fluorescence and T_1_ → S_0_ phosphorescence, further structural, energetic, and dynamic analyses must be performed for BF_2_-FORM-D. Based on the hypothesis that the non-radiative S_1_ → S_0_ relaxation occurs in an ultrashort 
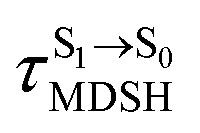
, the probability of the S_1_ → S_0_ fluorescence 
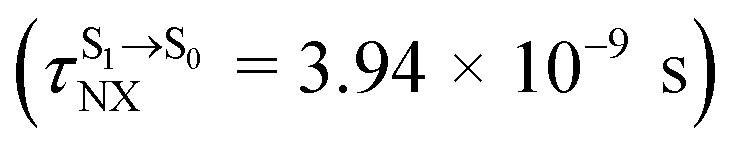
, S_1_/T_1_ ISC and T_1_ → S_0_ phosphorescence 
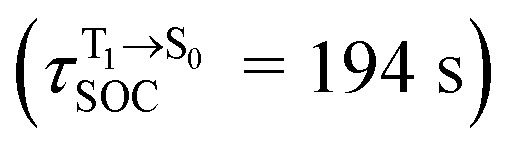
 could increase when 
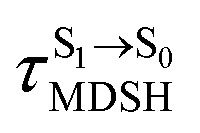
 is sufficiently long. Therefore, the factors that could affect the length of 
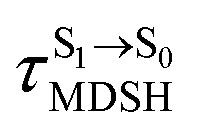
 were studied during NVE-MDSH simulations. Likewise, because the S_1_/T_1_ energy degeneration is one of the preconditions for the S_1_/T_1_ ISC and T_1_ → S_0_ phosphorescence, the factors affecting the duration of the S_1_/T_1_ energy degeneration were monitored during NVE-MDSH simulations.

The S_1_/T_1_ energy degenerations are clearly seen in Fig. 8c and d (yellow stripes), for which long S_1_/T_1_ degeneration times 
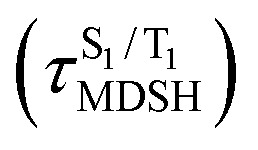
 are associated with non-L-ALM. The time evolutions of the S_1_ and T_1_ total energies also suggest that for non-L-ALM, each 
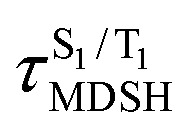
 could span between 
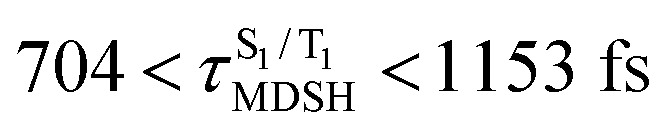
, compared with 
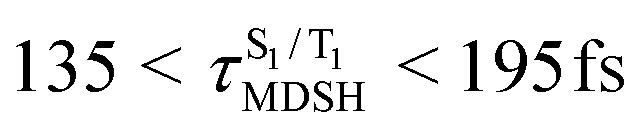
 for anti-L-ALM. In other words, the higher the probability of the non-L-ALM mode, the higher probability of the S_1_/T_1_ ISC and T_1_ → S_0_ phosphorescence.

**Fig. 9:**
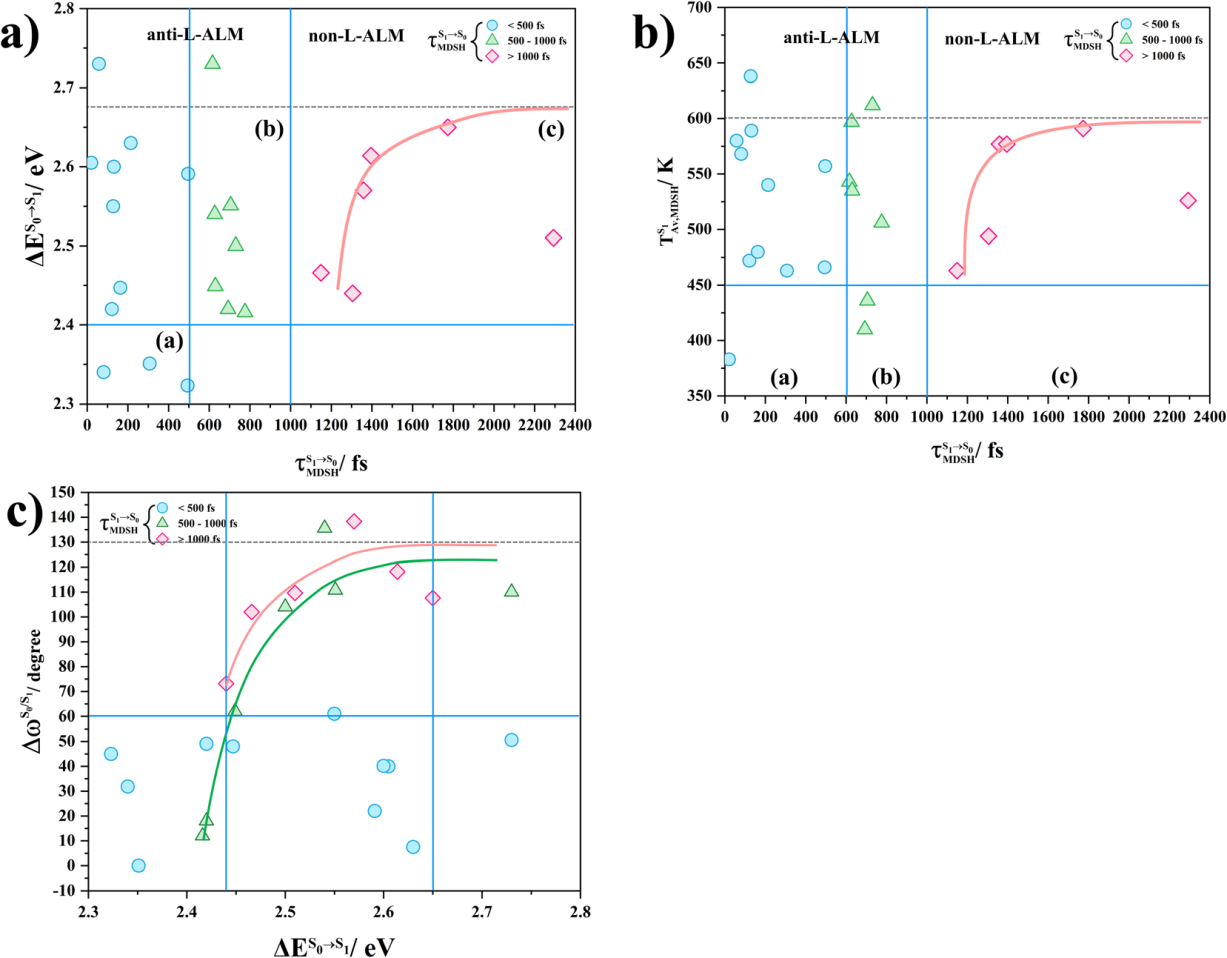
(a) Correlation between 
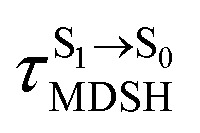
 and Δ*E*^S_0_→S_1_^. (b) Correlation between 
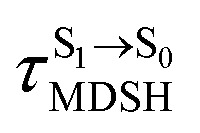
 and 
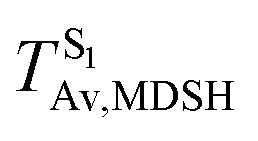
. (c) Correlation between 
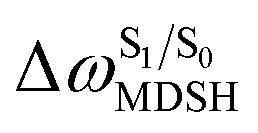
 and Δ*E*^S_0_→S_1_^. 
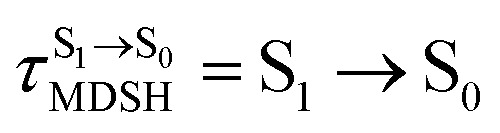
 surface hopping time; Δ*E*^S_0_→S_1_^ = S_0_ → S_1_ excitation energy; 
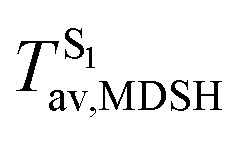
 = average temperature in the S_1_ state; 
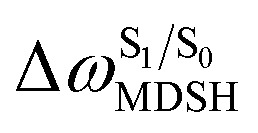
 = different between *ω*_1_ and *ω*_2_ at 
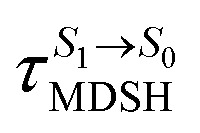
.

To study the effect of the absorbed radiation energy (the S_0_ → S_1_ excitation energy) on the non- and anti-L-ALM modes, the correlations among Δ*E*^S_0_→S_1_^ of the Wigner-sampled structures, average temperatures in the S_1_ state 
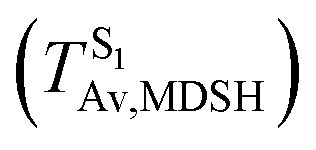
, S_1_ → S_0_ surface-hopping times 
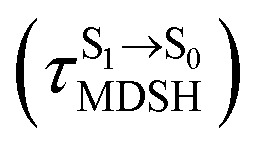
, and Δ*ω*_MDSH_ of the structures at 
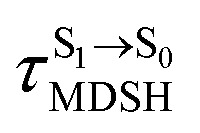

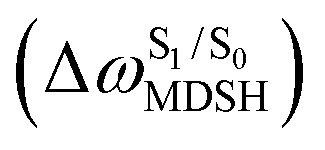
 obtained from all NVE-MDSH simulations are plotted in Fig. 9. The results show that for anti-L-ALM, 
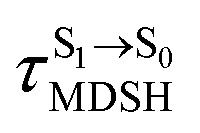
 can be categorized into two groups, namely, 
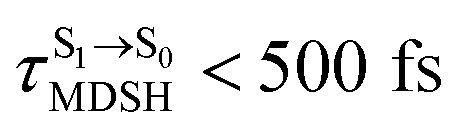
 and 
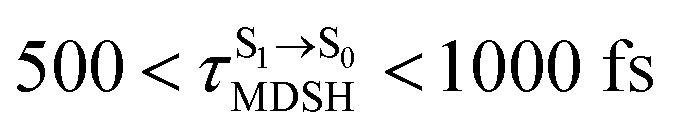
, whereas 
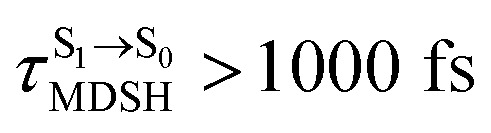
 is for non-L-ALM; these are indicated as (a), (b), and (c) in Fig. 9a, respectively.

It appears in Fig. 9a that anti-L-ALM (a) occurs exclusively in the absorbed radiation energy range of 2.30 < Δ*E*^S_0_→S_1_^ < 2.40 eV, whereas anti-L-ALM (a) and (b), and non-L-ALM (c) could be concurrently found when Δ*E*^S_0_→S_1_^ > 2.40 eV. The asymptotic behaviors of Δ*E*^S_0_→S_1_^, 
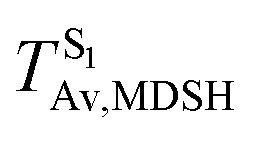
, and 
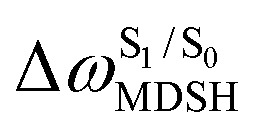
 in Fig. 9 suggest that to increase the probability of long S_1_ → S_0_ surface-hopping time, the absorbed radiation energy should be Δ*E*^S_0_→S_1_^ ≈ 2.67 eV or *λ*^abs^ ≈ 464 nm, corresponding to blue light source (Fig. 5b) with 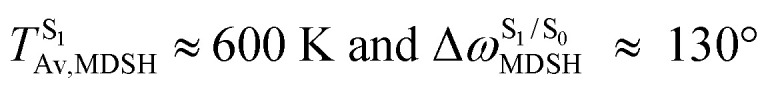.

These results suggest that the absorbed radiation energy and intramolecular librational motions govern the non-radiative S_1_ → S_0_ relaxation process and time, and non-L-ALM could increase the probability of fluorescence, S_1_/T_1_ ISC and phosphorescence. Because the TST results suggest that the S_1_/T_1_ ISC is thermodynamically favorable at *T*_s_ < 320 K and because the asymptotic average temperature for long S_1_ → S_0_ surface-hopping time is 
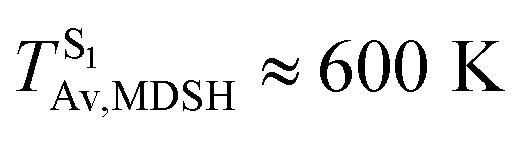
, to thermodynamically enhance the probability of photoluminescence, a temperature control mechanism is required.

## Conclusion

Photodynamic therapy (PDT) is a promising medical treatment for a range of human diseases, in which one of the key factors in its effectiveness is how well the photosensitizer can transfer photon energy to the target molecules. In this study, to improve the efficiency of photosensitizers for PDT, theoretical methods were used to study the photoluminescence mechanisms of BF_2_-formazanate dye (BF_2_-FORM) and its iodinated derivative (BF_2_-FORM-D) to investigate the heavy-atom effect. To complete this mechanistic study, complementary theoretical approaches were applied to investigate three important issues; (1) luminescence pathways in the S_1_ and T_1_ states, studied using DFT and TD-DFT methods, (2) kinetic and thermodynamic properties of the proposed pathways, studied using the TST method, and (3) time evolution and dynamics of the key processes, studied using NVE-MDSH simulations.

The DFT/B3LYP/6-311G and TD-DFT/B3LYP/6-311G results showed that in the S_0_ and T_1_ states, the equilibrium structures of BF_2_-FORM and BF_2_-FORM-D were similar, represented by bent and perfect planar structures, respectively, whereas in the S_1_ state, the equilibrium structure of BF_2_-FORM took a propeller structure and that of BF_2_-FORM-D a perpendicular structure. The HOMOs of the equilibrium structures in the S_0_ state were characterized by strong π character at the formazanate heterocyclic and phenyl rings, whereas electron density distribution in LUMOs was localized, and iodine substitutions at the phenyl rings led to a strong red-shift of Δ*E*^S_0_→S_1_^ close to the center of the visible light spectrum (green light with maximum intensity at 520 < *λ*^S_0_→S_1_^ < 532 nm).

The PESs for rotations of the dihedral angles *ω*_1_ and *ω*_2_ suggested two mechanisms for T_1_ → S_0_ transition: [T_1_ → S_0_]_1_ occurring immediately after S_1_/T_1_ ISC and [T_1_ → S_0_]_2_ occurring after S_1_/T_1_ ISC and T_1_ equilibrium structure relaxation. [T_1_ → S_0_]_2_ transition (Δ*E*^T_1_→S_0_^) is in the near IR range and close to the absorption energy for ^3^O_2_ (^3^∑_g_) → ^1^O_2_ (^1^Δ_g_). Because Δ*E*^S_0_→S_1_^ of BF_2_-FORM-D is closer to the center of the visible light spectrum and Δ*E*^S_1_→T_1_^ is significantly smaller than BF_2_-FORM, the iodinated derivative is anticipated to be a more effective luminophore, mainly owing to the heavy-atom effect. The RICC2/aug-cc-pVDZ (SOC-PT-CC2) calculations confirmed these findings and further suggested that the phosphorescence lifetime of [T_1_ → S_0_]_2_ of BF_2_-FORM-D is significantly longer than that of BF_2_-FORM.

Based on the PESs obtained using the DFT/B3LYP/6-311G, TD-DFT/B3LYP/6-311G, and NEB methods, TST calculations confirmed that [T_1_ → S_0_]_2_ of BF_2_-FORM-D is thermodynamically favorable below the spontaneous temperature (*T*_s_ = 320 K). The time evolutions of the dihedral angles *ω*_1_ and *ω*_2_ obtained from the analysis of NVE-MDSH simulations suggested that anti-L-ALM underlies ultrafast non-radiative S_1_ → S_0_ relaxation, whereas non-L-ALM could enhance the probability of S_1_/T_1_ ISC and photoluminescence. Therefore, to delay non-radiative S_1_ → S_0_ relaxation and increase the probability of photoluminescence, anti-L-ALM of the phenyl rings should be promoted. Analysis of the NVE-MDSH results suggested that the photoluminescence quantum yield could also be enhanced by varying the irradiation wavelength, for example, by using a blue light source to promote phosphorescence. Because efficient delivery of the photosensitizer to target cells is also a critical factor in PDT, our upcoming theoretical study will examine the photodynamic properties of BF_2_-FORM-D when incorporated into a specific nanostructure. These results will serve as a foundation for future theoretical and experimental research, to optimize and/or design effective PDT photosensitizers.

## Data availability

The data that support the findings of this study are available in the ESI† of this article.

## Conflicts of interest

There are no conflicts to declare.

## Supplementary Material

RA-014-D4RA02240H-s001
