# Supplementary material for: Photoluminescence mechanisms of BF2-formazanate dye sensitizers: a theoretical study
Source: RSC Adv. 2024 Jun 24;14(28):20081–92. doi: 10.1039/d4ra02240h (PMC11195527; doi:10.1039/d4ra02240h)
Supplement: RA-014-D4RA02240H-s001 [file RA-014-D4RA02240H-s001.pdf]

Electronic Supplementary Material (ESI) for RSC Advances.
This journal is © The Royal Society of Chemistry 2024

Electronic Supplementary Information (ESI)

Photoluminescence mechanisms of BF_2-formazanate dye sensitizers: A theoretical study

by

Parichart Suwannakham

Pannipa Panajapo

Phorntep Promma

Tunyawat Khrootkaew

Anyanee Kamkaew

and

*Kritsana Sagarik**

School of Chemistry

Institute of Science

Suranaree University of Technology

Nakhon Ratchasima 30000

Thailand

*corresponding author: *kritsana@sut.ac.th*

Tel./Fax: (6681) 8783994

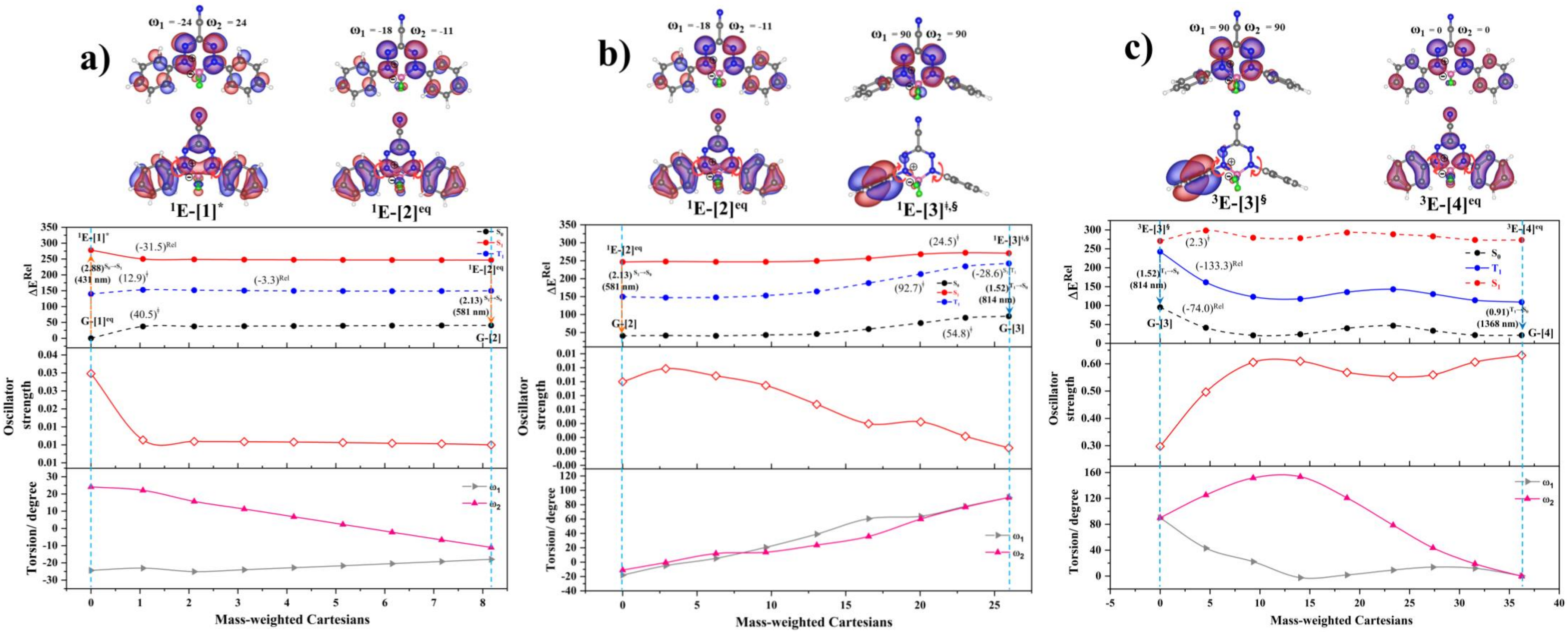

Figure S1 Potential energy surfaces for rotational motions of ω_1 and ω_2 in BF_2-FORM, obtained based on the hypothesized pathways in Figure 4, and the DFT/B3LYP/6-311G, TD-DFT/B3LYP/6-311G, and NEB methods. Dash lines are the potential energy curves computed using the geometries on the NEB potential energy curves (solid lines). a) $(I)^* \rightarrow (II)^*$. b) $(II)^* \rightleftharpoons (III)^{‡,§}$. c) $(III)^{‡,§} \rightarrow (IV)$.

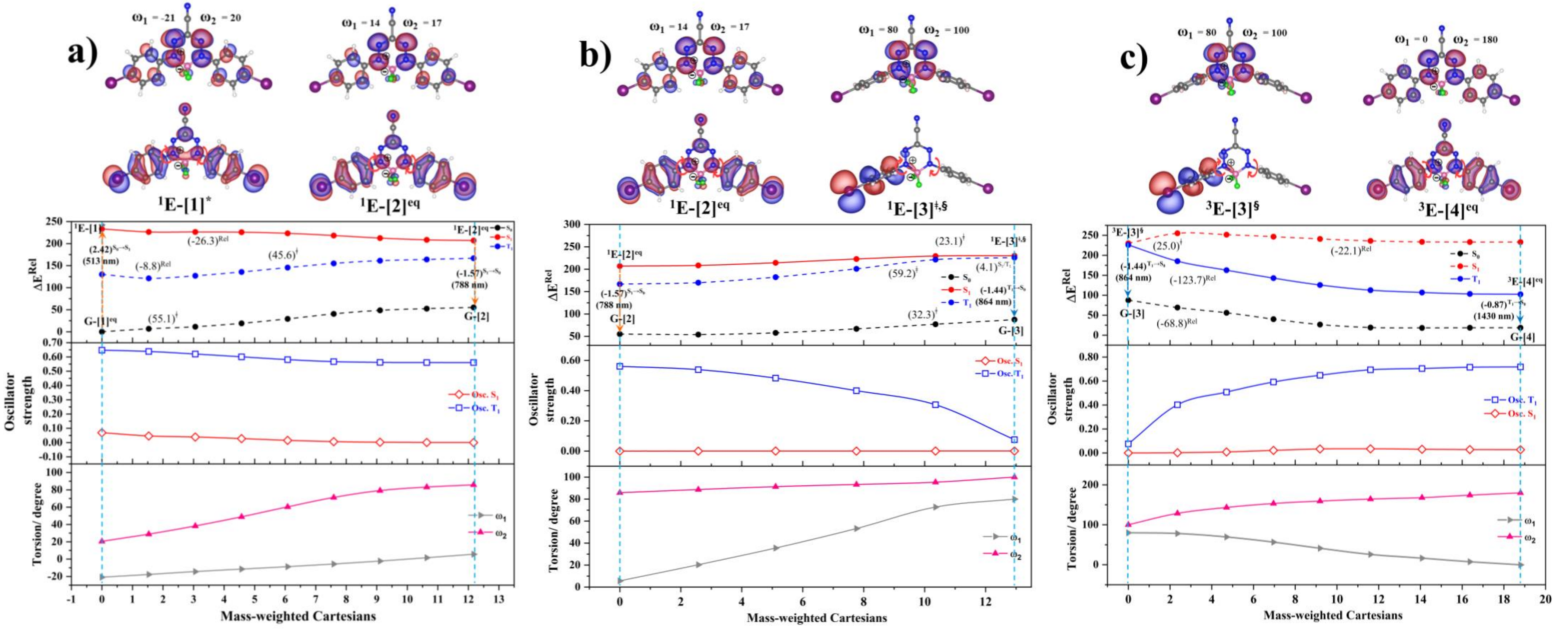

Figure S2 Potential energy surfaces for the rotational motions of ω_1 and ω_2 in BF_2-FORM-D, obtained based on the hypothesized pathways in Figure 4, and the DFT/B3LYP/6-311G, TD-DFT/B3LYP/6-311G, and NEB methods. Dash lines are the potential energy curves computed using the geometries on the NEB potential energy curves (solid lines). a) $(I)^* \rightarrow (II)^*$. b) $(II)^* \rightleftharpoons (III)^{‡,§}$. c) $(III)^{‡,§} \rightarrow (IV)$.

Table S1 Characteristic structures and energies of BF_2-FORM in the S_0, S_1 and T_1 states obtained from DFT/B3LYP/6-311G and TD-DFT/B3LYP/6-311G calculations.

**BF_2-FORM**	**TD-DFT**				**NX**			**RICC2**	
	$\Delta E^{S_0\rightarrow S_1}$	$\Delta E^{S_1\rightarrow S_0}$	$\Delta E^{S_1\rightarrow T_1}$	$\Delta E^{T_1\rightarrow S_0}$	$\Delta E_{NX}^{S_0\rightarrow S_1}$	$\Delta E_{NX}^{S_1\rightarrow S_0}$	$\tau_{NX}^{S_1\rightarrow S_0}$	$\Delta E_{SOC}^{T_1\rightarrow S_0}$	$\tau_{SOC}^{T_1\rightarrow S_0}$
**G-[1]^{eq}** ω_1 = -24 ω_2 = 24 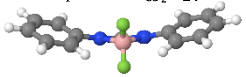 E^{Tot} = -1037.31086	2.88	-	-	-	2.71	-	2.95×10^{-9}	-	-
**^{1}E-[2]^{eq}** ω_1 = -18 ω_2 = -11 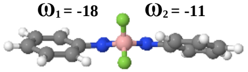 E^{Tot} = -1037.21702	-	-2.13	-	-	-	-2.46	8.88×10^{-7}	-	-
**^{1}E-[3]§** ω_1 = 90 ω_2 = 90 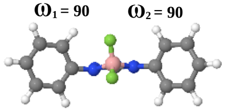 E^{Tot} = -1037.20770	-	-1.82	-0.30	-1.52 (P_1)	-	-	-	-1.94 (P_1)	0.36
**^{3}E-[4]^{eq}** ω_1 = 0 ω_2 = 0 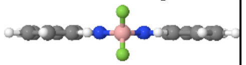 E^{Tot} = -1037.26944	-	-0.91	-	-0.91 (P_2)	-	-0.91	1.13×10^{-6}	-1.05 (P_2)	131

E^{Tot} = total energy in au; $\Delta E^{S_0\rightarrow S_1}$ = excitation energy in eV; $\Delta E_{NX}^{S_0\rightarrow S_1}$ = excitation energy obtained based on 200 Wigner sampled structures; ω_1 and ω_2 = dihedral angles defined in Figure 2; $\Delta E^{T_1\rightarrow S_0}$ = singlet-triplet energy gap on the potential energy surface; […]^{eq} = equilibrium structure; […]§ = structure at the S_1/T_1 intersection; $\tau_{NX}^{S_1\rightarrow S_0}$ and $\tau_{SOC}^{T_1\rightarrow S_0}$ = fluorescence and phosphorescence lifetimes in s, respectively; $\Delta E_{SOC}^{T_1\rightarrow S_0}$ = singlet-triplet energy gap obtained from SOC-PT-CC2/aug-cc-pVDZ calculations.

Table S2 The characteristic structures and energies of BF_2-FORM-D in the S_0, S_1 and T_1 states obtained from DFT/B3LYP/6-311G and TD-DFT/B3LYP/6-311G calculations.

**BF_2-FORM-D**	**TD-DFT**				**NX**			**RICC2**	
	$\Delta E^{S_0\rightarrow S_1}$	$\Delta E^{S_1\rightarrow S_0}$	$\Delta E^{S_1\rightarrow T_1}$	$\Delta E^{T_1\rightarrow S_0}$	$\Delta E_{NX}^{S_0\rightarrow S_1}$	$\Delta E_{NX}^{S_1\rightarrow S_0}$	$\tau_{NX}^{S_1\rightarrow S_0}$	$\Delta E_{SOC}^{T_1\rightarrow S_0}$	$\tau_{SOC}^{T_1\rightarrow S_0}$
**G-[1]^{eq}** $\omega_1 = -21$ $\omega_2 = 21$ 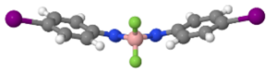 E^{Tot} = -1058.87541	2.42	-	-	-	2.32	-	3.94×10^{-9}	-	-
**^{1}E-[2]^{eq}** $\omega_1 = 5$ $\omega_2 = 86$ 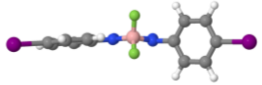 E^{Tot} = -1058.79661	-	-1.57	-	-	-	-1.65	8.49×10^{-7}	-	-
**^{1}E-[3]§** $\omega_1 = 80$ $\omega_2 = 100$ 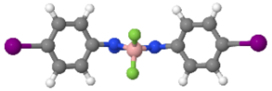 E^{Tot} = -1058.78779	-	-1.48	-0.04	-1.43 (P_1)	-	-	-	-1.93 (P_1)	0.33
**^{3}E-[4]^{eq}** $\omega_1 = 0$ $\omega_2 = 0$ 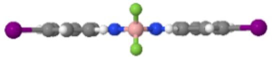 E^{Tot} = -1058.83644	-	-0.88	-	-0.87 (P_2)	-	-0.94	5.20×10^{-8}	-0.98 (P_2)	194

E^{Tot} = total energy in au; $\Delta E^{S_0\rightarrow S_1}$ = excitation energy in eV; $\Delta E_{NX}^{S_0\rightarrow S_1}$ = excitation energy obtained based on 200 Wigner sampled structures; ω_1 and ω_2 = dihedral angles defined in Figure 2; $\Delta E^{T_1\rightarrow S_0}$ = singlet-triplet energy gap on the potential energy surface; […]^{eq} = equilibrium structure; […]§ = structure at the S_1/T_1 intersection; $\tau_{NX}^{S_1\rightarrow S_0}$ and $\tau_{SOC}^{T_1\rightarrow S_0}$ = fluorescence and phosphorescence lifetimes in s, respectively; $\Delta E_{SOC}^{T_1\rightarrow S_0}$ = singlet-triplet energy gap obtained from SOC-PT-CC2/aug-cc-pVDZ calculations.

Table S3 Thermodynamics and kinetics of the photoluminescence pathway (Figure 4) for BF_2-FORM. Rate constants, temperatures and energies are in s^{-1}, K and kJ/mol, respectively; $\Delta E^{\ddagger}$ = energy barrier; $\Delta E^{\ddagger,ZPC}$ = zero-point energy-corrected barrier, obtained by including the zero-point correction energy to the energy barrier obtained from the NEB method ($\Delta E^{\ddagger}$); $\Delta H^{\ddagger}$ = activation enthalpy; $\Delta S^{\ddagger}$ = activation entropy; $\Delta G^{\ddagger}$ = activation Gibbs free energies; T_c = crossover temperature; T = temperature; $k_{f/r}^{Q\text{-}vib}$ = rate constant obtained with quantized vibrations including the zero-point vibrational energy; f/r = forward or reverse direction.

Table S3

Reaction Pathway	$\Delta E^{‡}$	$\Delta E^{‡,ZPC}$	$\Delta H^{‡}$	T_c	T	$k_{f/r}^{Q\text{-}vib}$	$\Delta G^{‡}$	$\Delta S^{‡}$
$^{1}E\text{-}[1]^{*} \leftarrow {}^{1}E\text{-}[2]^{eq}$	31.5	20.3	23.8	372	300	3.24×10^{9}	18.87	1.63×10^{-2}
					330	7.51×10^{9}	18.71	1.53×10^{-2}
					413	4.18×10^{10}	18.27	1.34×10^{-2}
					550	2.47×10^{11}	17.55	1.13×10^{-2}
$^{1}E\text{-}[2]^{eq} \rightarrow {}^{1}E\text{-}[3]^{‡,§}$	24.4	20.7	21.8	5	300	5.85×10^{8}	23.14	-4.51×10^{-3}
					330	1.29×10^{9}	23.55	-5.36×10^{-3}
					413	6.27×10^{9}	24.78	-7.25×10^{-3}
					550	3.10×10^{10}	27.03	-9.54×10^{-3}
$^{3}E\text{-}[3]^{§} \leftarrow {}^{3}E\text{-}[4]^{eq}$	133.3	127.1	129.3	42	300	5.26×10^{-10}	126.78	8.40×10^{-3}
					330	5.79×10^{-8}	126.82	7.51×10^{-3}
					413	7.13×10^{-4}	126.99	5.60×10^{-3}
					550	9.03×10^{0}	127.45	3.38×10^{-3}

Table S4 Thermodynamics and kinetics of the photoluminescence pathway (Figure 4) for BF_2-FORM-D. Rate constants, temperatures and energies are in s^{-1}, K and kJ/mol, respectively; $\Delta E^{\ddagger}$ = energy barrier; $\Delta E^{\ddagger,ZPC}$ = zero-point energy-corrected barrier, obtained by including the zero-point correction energy to the energy barrier obtained from the NEB method ($\Delta E^{\ddagger}$); $\Delta H^{\ddagger}$ = activation enthalpy; $\Delta S^{\ddagger}$ = activation entropy; $\Delta G^{\ddagger}$ = activation Gibbs free energies; T_c = crossover temperature; T = temperature; $k_{f/r}^{Q\text{-}vib}$ = rate constant obtained with quantized vibrations including the zero-point vibrational energy; f/r = forward or reverse direction.

Table S4

Reaction Pathway	ΔE‡	ΔE^{‡,ZPC}	ΔH‡	T_c	T	$k_{f/r}^{Q\text{-}vib}$	ΔG‡	ΔS‡
**^{1}E-[1]* ← ^{1}E-[2]^{eq}**	26.3	32.9	30.4	4	300	2.26×10^{4}	48.49	-6.03×10^{-2}
					330	6.92×10^{4}	50.52	-6.10×10^{-2}
					413	6.40×10^{5}	56.29	-6.28×10^{-2}
					550	5.74×10^{6}	66.34	-6.53×10^{-2}
**^{1}E-[2]^{eq} → ^{1}E-[3]‡,§**	23.1	30.0	26.9	4	300	2.45×10^{4}	48.29	-7.12×10^{-2}
					330	6.64×10^{4}	50.64	-7.18×10^{-2}
					413	4.78×10^{5}	57.29	-7.36×10^{-2}
					550	3.31×10^{6}	68.86	-7.62×10^{-2}
**^{3}E-[3]§ ← ^{3}E-[4]^{eq}**	123.7	118.7	120.7	134	300	1.08×10^{-8}	119.25	4.74×10^{-3}
					330	8.68×10^{-7}	119.40	3.87×10^{-3}
					413	5.70×10^{-3}	120.86	1.97×10^{-3}
					550	3.84×10^{1}	120.83	-2.81×10^{-4}
